# Reasoning-based LLMs surpass average human performance on medical social skills

**DOI:** 10.1038/s41598-025-20496-7

**Published:** 2025-10-17

**Authors:** Khalid Ibraheem Alohali, Laura Asaad Almusaeeb, Abdulaziz Abdulrahman Almubarak, Ahmad Ibraheem Alohali, Ruaim Abdullah Muaygil

**Affiliations:** 1 College of Medicine, King Saud University, Riyadh, 11461 Saudi Arabia; 2 College of Computer and Information Sciences, King Saud University, Riyadh, 11461 Saudi Arabia; 3 Associate Professor of Healthcare Ethics, Department of Medical Education, College of Medicine, King Saud University, Riyadh, Saudi Arabia

**Keywords:** Artificial intelligence, Large language models (LLMs), Social skills, Medical ethics, Medical education, Machine learning, Medical ethics

## Abstract

A significant portion of medical licensing examinations assesses key social skills such as communication, ethics, and professionalism, which are vital for quality patient care. Artificial intelligence (AI) has been increasingly integrated into healthcare systems in recent years, raising concerns among regulators, providers, and patients regarding AI’s capacity to handle complex, human-centered scenarios. Previous work has shown that large language models (LLMs) like GPT-3.5 and GPT-4 perform well on social skills questions from the United States Medical Licensing Examination (USMLE). However, newer models like GPT-4o, Gemini 1.5 Pro, and o1 have been introduced, with the latter designed to mimic human thinking through a “chain of thought” reasoning, unlike other LLMs that provide instantaneous answers. The impact of reasoning on LLMs’ ability to navigate scenarios requiring social skills remains unclear. Here, we evaluate five LLMs: GPT-4, GPT-4o, Gemini 1.5 Pro, and o1-preview, and its full version, o1; using forty USMLE-style social skills questions from the UWORLD question bank covering several categories: communication & interpersonal skills, healthcare policy & economics, system-based practice & quality improvement, and medical ethics & jurisprudence. After each LLM answered, it was subjected to an “Are you sure?” follow-up prompt to test consistency. Our results show that o1, the reasoning model, came out on top with 39 out of 40 correct final answers (97.5%). GPT-4o and Gemini 1.5 Pro (87.5%) tied in second place, followed by o1-preview (77.5%) and lastly GPT-4 (75%). All LLMs surpassed the UWORLD question bank’s 64% average. Domain-specific analysis revealed that despite having equal overall scores, GPT-4o and Gemini 1.5 Pro -developed by two different companies- had varying strengths. GPT-4o demonstrated its greatest strengths in communication & interpersonal skills and patient safety, while Gemini 1.5 Pro achieved perfect scores in healthcare policy & economics, system-based practice & quality improvement, and medical ethics & jurisprudence. Although o1-preview demonstrated strong initial performance, its inconsistency under skepticism; changing answers frequently, primarily to incorrect ones, reduced its overall ranking from second to fourth. This phenomenon was not observed in any other model, including the final o1 release, which maintained consistent, high-level performance. These findings, along with prior work, highlight the potential of LLMs to demonstrate effectiveness at answering knowledge-based social skills questions in a medical context, sometimes surpassing average human performance. As LLMs continue to grow in size and sophistication, their performance is expected to improve further. In particular, the strong performance of reasoning-based LLMs suggests that such architectures hold significant promise for advancing AI’s role in socially oriented tasks. These results demonstrate the growing potential for reasoning-based LLMs to complement and enhance clinical training, medical education, and patient care.

**Supplementary Information:**

The online version contains supplementary material available at 10.1038/s41598-025-20496-7.

## Introduction

**Fig. 1:**
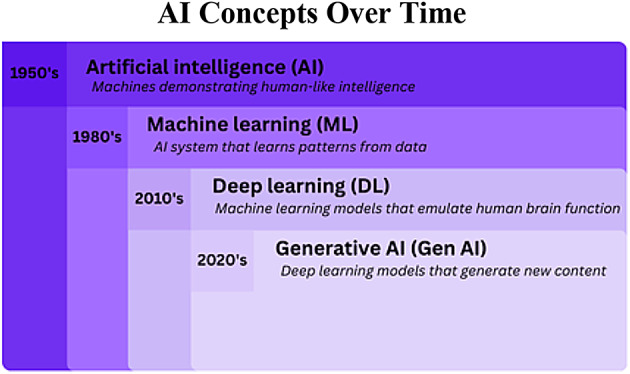
“A simple way to think About AI is as a series of nested or derivative concepts that have emerged over more than 70 years.” This figure was adapted from *IBM*^1^.

Artificial Intelligence (AI) can be broadly defined as the artificial simulation of human intelligence, encompassing capabilities such as learning, problem-solving, comprehension, decision-making, creativity, and autonomy. AI has been gradually evolving since the 1950 s when Alan Turing, the so-called “father of computer science,” published his breakthrough paper inquiring, “Can Machines Think?” While an extensive review of AI’s history and its various types falls outside the scope of this paper, it is essential for non-specialists exploring AI applications in their respective fields to clearly understand fundamental AI concepts and terminology, as these terms are frequently intermixed. Figure 1, illustrates the key concepts and types of AI that have emerged over the past 70 years^1^.

In recent years, AI has become a trending topic across many fields since OpenAI’s ChatGPT, based on the large language model (LLM) GPT-3.5 was first released to the public in 2022. ChatGPT marked the beginning of an era where AI’s multi-industry potential has become more evident than ever. By 2024, hundreds of new models were developed globally, with AI being integrated into the workflows of numerous businesses and organizations. Millions of students, professionals, and researchers now utilize AI tools daily^2^. Table 1 lists the LLMs discussed in this study, including OpenAI’s “o1”, an LLM claimed to perform complex reasoning and take time to think before responding, “much like a person would.”^3–8^.

Among the fields where AI can play a pivotal role is healthcare. Healthcare spending accounts for at least 10% of gross domestic product (GDP) in most developed countries^9^, indicating great room for innovation and optimization. While the idea of incorporating AI in healthcare is promising, globally legislative bodies are being established to ensure the utmost quality and safety for patients, playing a significant role in regulating AI. Interestingly, these legislative bodies not only regulate drugs and medical technology, they also regulate the practice of health professionals such as physicians.

Although AI has demonstrated capability within the scientific and predictive aspects of medicine, its capacity to handle scenarios requiring social skills within a clinical environment, where human empathy and intuition are crucial, remains debatable^10–12^.

Recently, LLMs have shown remarkable potential in passing standardized medical examinations and enhancing medical education^10^. Medical students globally must complete country-specific medical licensing examinations before entering clinical practice. In many regions, these exams determine eligibility and assess competency and competitiveness for medical residency programs. Examples include the United States Medical Licensing Examination (USMLE)^13^, the Medical Council of Canada Qualifying Examination (MCCQE)^14^, the United Kingdom’s Medical Licensing Assessment (MLA)^15^, and Japan’s National Medical Licensing Examination (NMLE)^16^. Scholarly evidence suggests that test results can predict future clinical performance; for example, a recent study showed that higher USMLE scores among providers correlated with lower in-hospital mortality rates and shorter lengths of stay^17^.

While these examinations primarily focus on evaluating candidates’ knowledge of basic and clinical medical sciences, a substantial aspect of their evaluation targets non-medical skills, including ethics, communication, and professionalism. Although the terms used to describe this set of skills may differ contextually, they often overlap; examples of terms used in the literature include soft^18^, social^13,19,20^, and non-technical skills^21^. *The Textbook of Patient Safety and Clinical Risk Management* defines non-technical skills as “a constellation of cognitive and social skills, exhibited by individuals and teams, needed to reduce error and improve human performance in complex systems^21^. USMLE questions that test these skills fall under the “social sciences” discipline, which encompasses communication and interpersonal skills, medical ethics and jurisprudence, systems-based practice and patient safety, and health care policy and economics^13,19,20^. To maintain consistency, this study will use the term social skills, as it aligns with the terminology utilized by the USMLE, the source of the assessment tool. However, this does not imply it is the most precise term, and the nuanced distinctions between these terms must be appreciated.

Nevertheless, a pioneering study published in *Nature* in 2023 compared the performance and consistency of ChatGPT (GPT-3.5) and GPT-4 on USMLE soft skills assessments using a combination of USMLE’s publicly released retired questions and AMBOSS’s question bank, a widely used medical knowledge platform. The study found that GPT-4 was superior to GPT-3.5 in performance and consistency. It concluded that LLMs can significantly enhance human abilities in healthcare, particularly in tasks requiring empathy and sound judgment^18^.

Since the publication of that study, several new and enhanced LLMs have been released; however, their performance in social skills within a clinical context has yet to be systematically assessed. Table 1^3–8^ covers all of the models covered in this study. GPT-3.5, GPT-4, and GPT-4o are LLMs developed by OpenAI; GPT-3.5 (2022)^3^ originally powered ChatGPT; GPT-4 (2023)^4^ added multimodal image input; and GPT-4o “omni” (2024)^5^ is natively multimodal for real-time text, vision, and audio. Another LLM covered in this study is Google’s Gemini 1.5 Pro (2024)^8^. It is a mid-size, multimodal “Mixture-of-Experts” model with a standard 128k context window.

Among these new models is OpenAI’s “o1,” a series of LLMs designed to perform chain-of-thought reasoning before responding. A preview version, o1-preview, was released in September 2024, followed by the full version two months later^6,22^. While LLM scaling laws indicate that larger models tend to perform better^23,24^, specific properties, such as chain-of-thought reasoning, may influence social skills performance in unpredictable ways. This study aims to evaluate the proficiency of OpenAI’s GPT-4, GPT-4o, o1-preview, o1, and Google’s Gemini 1.5 Pro in USMLE’s Step 1 social science section. In addition, we specifically would like to uncover whether reasoning significantly impacts an LLM’s performance in medical social skills assessments. This research contributes novel insights by testing a broader range of LLMs, utilizing diverse question banks, and categorizing questions to allow for practical extrapolations and detailed sub-analyses. Additionally, this study incorporates performance data from past users, facilitating a comparative analysis of LLM performance against that of medical students and practicing physicians.

**Table 1:** Overview of LLMs discussed in this Study3–8.

Model	Developer	Year Released	Official Website
GPT-3.5	Open AI	2022	^3^
GPT-4		2023	^4^
GPT-4o		2024	^5^
o1-preview		Sep 2024	^6^
o1		Dec 2024	^7^
Gemini 1.5 Pro	Google	2024	^8^

## Methods

### Large Language models

Beginning in September 2024, our study evaluated the performance and consistency of four widely used LLMs: GPT-4, GPT-4o, o1-preview, and Gemini 1.5 Pro. Because GPT-4 had already been assessed in a previous study^18^, it served as a benchmark for comparing reproducibility between the earlier findings and our new results. During the final stages of our manuscript preparation in December 2024, o1-preview was superseded by its full release (o1), which we incorporated into our assessment to enhance the practical significance of our study.

### Question sources and data extraction

The models were asked a total of forty-five questions. All were multiple-choice questions testing USMLE Step 1 social skills concepts. They were extracted from two sources: Firstly, five official USMLE Step 1 retired questions were adopted from the previous study for benchmarking purposes. Secondly, forty questions or “a block” of questions were randomly extracted from UWORLD’s social sciences section, which consists of more than 100 questions. UWORLD is often regarded as the most realistic USMLE Step 1 question bank^25,26^. Randomization was essential to ensure a variety of question types and complexities; the first 40 questions meeting our inclusion criteria in the random sequence were included. In addition, because UWORLD provides average correct response rates based on its extensive history of previous users, predominantly first and second-year medical students. These data allowed for a new layer of comparative analysis of LLM performance against typical test-takers.

### Inclusion and exclusion criteria

Questions from both sources were included, and each question was individually assessed for eligibility by three members of the research team. Questions requiring a high level of scientific medical knowledge to answer, as determined by at least two of three researchers, were replaced with other randomly selected questions that did not require such knowledge.

**Fig. 2:**
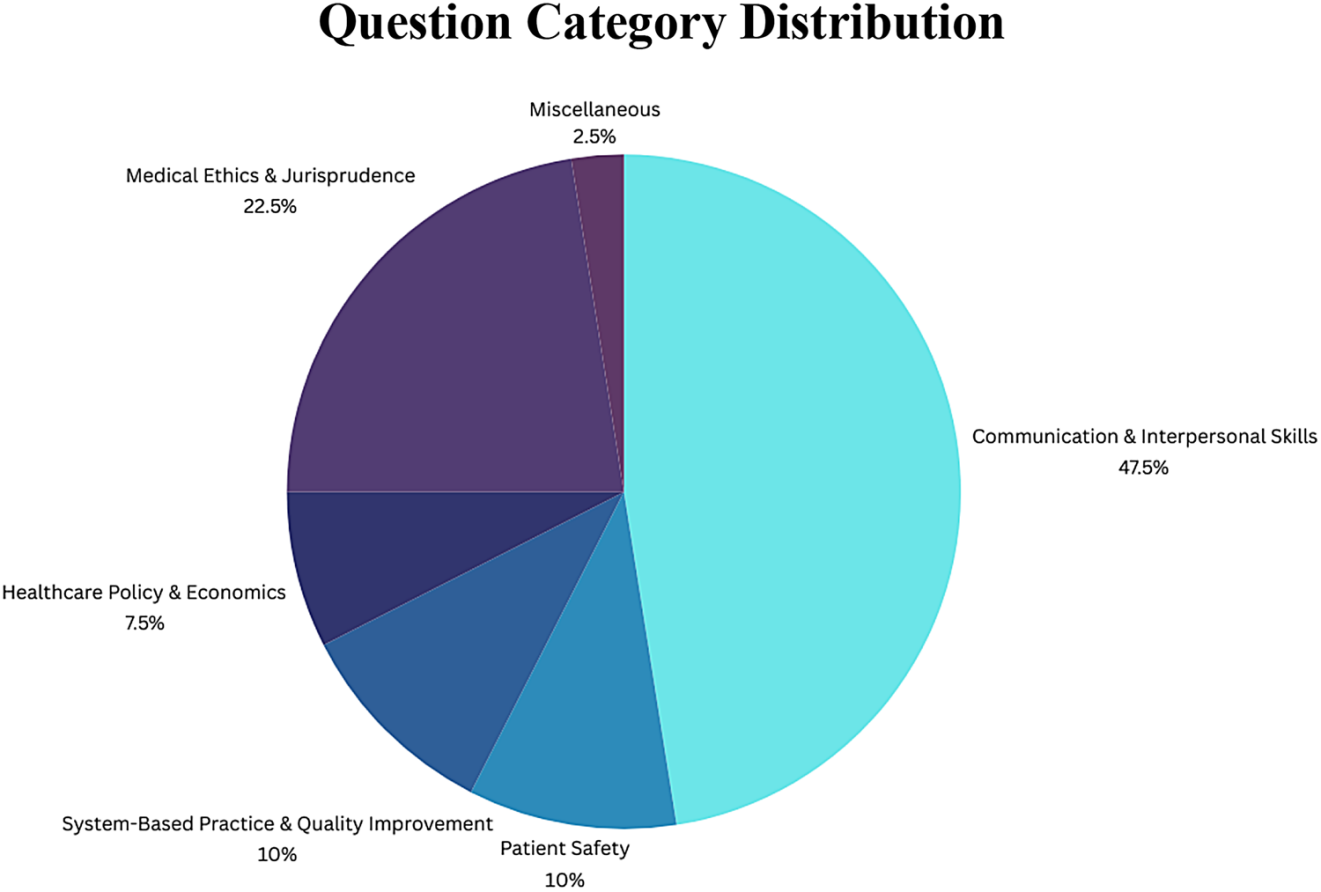
Breakdown of the tested categories from the 40-question set extracted from the UWORLD question bank. The most tested categories were communication and interpersonal skills (19), medical ethics & jurisprudence (9), system-based practice & quality improvement (4), patient safety (4), healthcare policy & economics (3), and miscellaneous (1).

### Question categories

Figure 2 illustrates the tested category of each of the forty questions extracted from the UWORLD question bank. The most tested categories, descending, were communication and interpersonal skills (19), medical ethics and jurisprudence (9), system-based practice and quality improvement (4)/patient safety (4), healthcare policy and economics (3), and miscellaneous(1).

### Prompt

The prompt followed the same procedure as the original benchmarking study’s two-step prompt structure: First, presenting the question alongside multiple-choices directly copied from the question source^18^. Second, After the model responded, a follow-up question was asked, “Are you sure?”. This serves two purposes: first, to determine whether these models are skeptical enough to reconsider an incorrect choice, and second, to assess their confidence in upholding correct answers when their certainty was questioned. The follow-up therefore functions as a skepticism-stress test rather than an objective measure of reliability. Prompts were done by a single researcher consistently. All LLM responses were stored in an Excel sheet (Supplementary Table 1). Because the prompting of each item consists of two steps: (i) the verbatim question-and-option text shown in Supplementary Tables 1 and (ii) the single follow-up line “Are you sure?”, readers can reconstruct the exact wording directly from Supplementary Table 1. All models were accessed via their default respective ChatGPT/Gemini platform with no adjustment to temperature, top_p, top_k, or other generation settings; default values were used to reflect a typical user experience. No fine-tuning or custom instructions were applied beyond what is embedded in the ChatGPT/Gemini platform.

### Statistical analysis

We evaluated two pre-specified hypotheses: (i) whether the reasoning-based model (o1) surpasses the UWORLD user benchmark (64%) using a one-sided exact binomial test (*n* = 40), and (ii) whether o1 exceeds the best non-reasoning LLM using a two-sided Fisher exact test on 2 × 2 correct/incorrect counts. GPT-4o and Gemini 1.5 Pro tied at 35/40; we report the o1 vs. GPT-4o comparison and note that o1 vs. Gemini yields the identical Fisher table and p-value. Family-wise Type I error across the two primary comparisons was controlled with a Bonferroni adjustment (α_adj = 0.025). In addition, to quantify chance-corrected within-item self-agreement, we computed unweighted Cohen’s κ between each model’s initial answer and its post–“Are you sure?” answer across the 40 items (κ = 0 indicates chance; κ = 1 perfect agreement). Analyses were performed in R (v4.5.1) using base functions and the irr package; full outputs, inputs, and code are provided in Supplementary Tables 2–4 and the provided R code.

## Results

### Overall performance

**Fig. 3:**
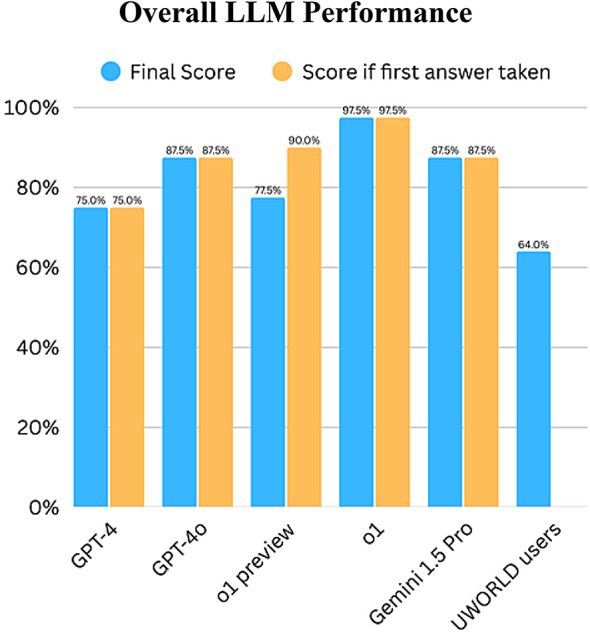
This bar chart illustrates the final scores of LLMs on the 40-question set, compared with the scores if the first answer, before the skepticism prompt, had been taken. Final accuracies were: o1 (97.5%), GPT-4o (87.5%), Gemini 1.5 Pro (87.5%), o1-preview (77.5%), and GPT-4 (75%); the UWORLD users’ average was 64%. Aside from o1-preview, no LLM changed any answers. o1-preview changes resulted in a net-negative effect. If it wasn’t for the changes, o1-preview would have scored the second highest (90%), exceeding GPT-4o and Gemini 1.5 Pro (both 87.5%).

We evaluated five large language models (LLMs): GPT-4, GPT-4o, Gemini 1.5 Pro, o1-preview, and o1, across five previously used USMLE Step 1 social sciences questions for benchmarking purposes and forty unique. To simulate real-world situations, we only considered each model’s final response after prompting, “Are you sure?” in our overall performance analysis. Figure 3 summarizes the overall performance relative to the average performance of UWORLD question bank users.

Regarding the first five questions, GPT-4 provided the same answers as the earlier study, indicating reproducibility and consistency within the same LLM among users at different times.

For the forty unique questions, the LLMs demonstrated the following overall performances: The highest-performing LLM was o1, which answered 39 of 40 questions correctly (97.5%). GPT-4o and Gemini 1.5 Pro both scored 87.5%, followed by o1-preview (77.5%) and GPT-4 (75%). Notably, o1’s score represents a substantial improvement over its earlier preview version, indicating rapid model adjustment. These LLM scores are juxtaposed to the UWORLD users’ average correct score of 64% on the same 40 questions (Fig. 3), which is lower than any LLM tested in this study. o1’s accuracy (39/40; 97.5%) significantly exceeded the UWORLD benchmark of 64% (exact binomial *p* = 4.15 × 10⁻⁷; Bonferroni-adjusted *p* = 8.30 × 10⁻⁷). In head-to-head comparison with the best non-reasoning LLMs (GPT-4o and Gemini 1.5 Pro, each 35/40), the 4 out 40 difference was not statistically significant on this item set (two-sided Fisher exact *p* = 0.201; Bonferroni-adjusted *p* = 0.401); because GPT-4o and Gemini tied, the Fisher result is identical for both.

Communication & Interpersonal Skills (19): o1 & GPT-4o (94.74%), Gemini 1.5 Pro & o1-preview (78.95%), GPT-4 (73.68%), UWORLD users (64.89%) Medical Ethics & Jurisprudence (9): o1 & Gemini 1.5 Pro (100.00%), o1-preview (88.89%), GPT-4o & GPT-4 (77.78%), UWORLD users (64.11%) System-Based Practice & Quality Improvement (4): o1 & Gemini 1.5 Pro (100.00%), GPT-4o & GPT-4 (75.00%), UWORLD users (72.75%), o1-preview (50.00%) Patient Safety (4): o1 & GPT-4o (100.00%), GPT-4, Gemini 1.5 Pro, & o1-preview (75.00%), UWORLD users (58.75%) Healthcare Policy & Economics (3): o1 & Gemini 1.5 Pro (100.00%), GPT-4o, GPT-4, & o1-preview (66.67%), UWORLD users (60.00%) Miscellaneous (1): All LLMs tested(100.00%), UWORLD users (78.00%)

**Fig. 4:**
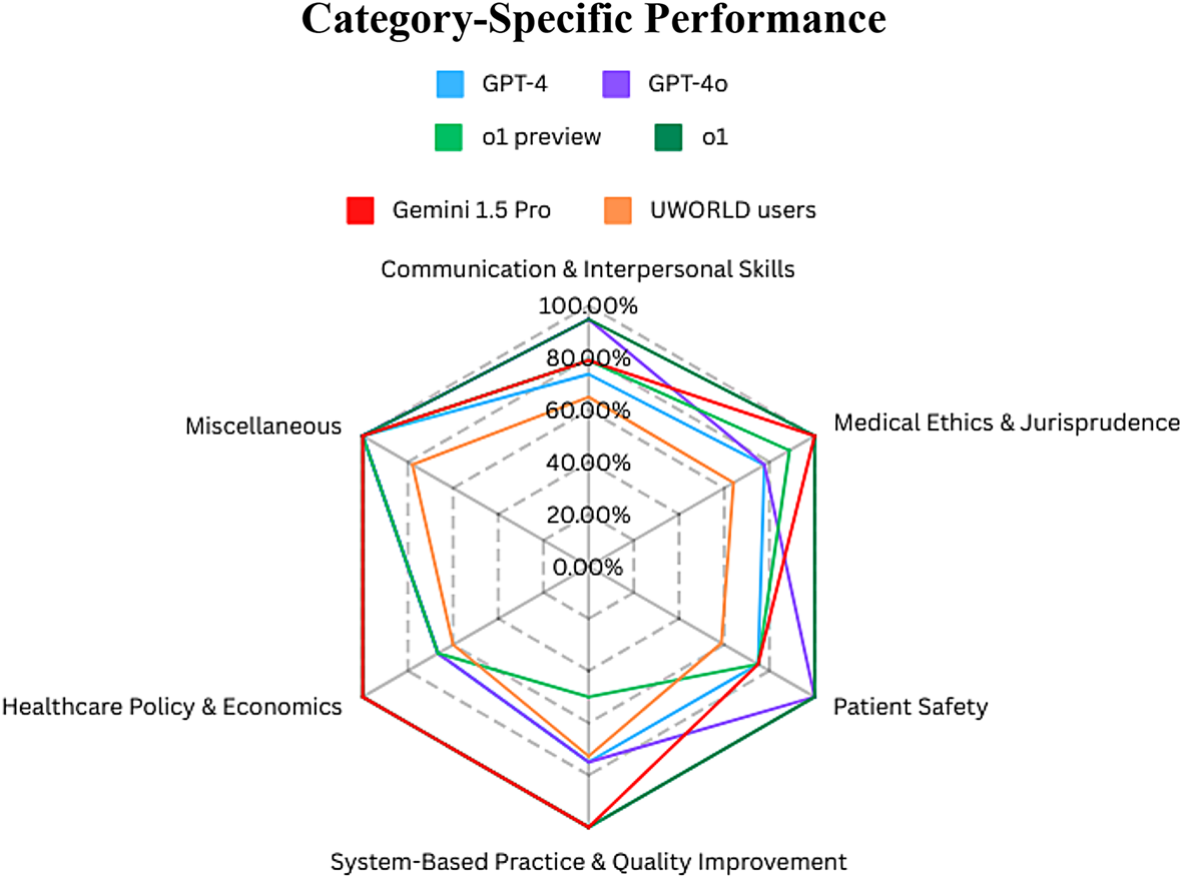
The radar chart illustrates the performance of GPT-4, GPT-4o, o1-preview, and Gemini 1.5 Pro across six categories; Performance ordered by number of questions per category and ranked by highest to lowest scores.

### Category-specific performance

The categorized questions allowed for a detailed sub-analysis of the specific social skills in which each LLM excelled. It also allowed for comparing these performances with UWORLD users’ average correct responses within that domain. As shown in *Fig. 4*, o1, the reasoning model had perfect scores in all categories, with its only mistake being in the communication & interpersonal skills. Notably, the LLMs that shared the second place, GPT-4o and Gemini 1.5 Pro (both at 88%), demonstrated different areas of strength. GPT-4o achieved near-perfect scores in communication & interpersonal skills (94.74%) and patient safety (100%), while Gemini 1.5 Pro excelled with perfect scores in healthcare policy & economics, system-based practice & quality improvement, and medical ethics & jurisprudence (100% in all three categories).

In comparison, GPT-4 performed well but lagged slightly behind, scoring 73.68% in communication & interpersonal skills and 75% in patient safety. o1-preview performed best within the medical ethics & jurisprudence (88.89%) and healthcare policy & economics (66.67%). However, it ranked last in system-based practice & quality improvement (50%), the only category in which UWORLD users (72.75%) outperformed an LLM.

### The impact of skepticism on LLM performance and Self-Agreement

Figure 3 provides insight into the effects of skepticism on the performance of LLMs. As shown in Fig. 3, o1-preview demonstrated the second highest initial accuracy, scoring 90% if the first answer was considered without any modifications. However, after the “Are you sure?” prompt, the o1-preview dropped to 78%, making it the only model that changed its answers following skepticism. In contrast, o1, GPT-4, GPT-4o, and Gemini 1.5 Pro displayed consistent performance, as they never altered their initial answers, retaining final scores of 75%, 87.5%, and 87.5%, respectively. Taking a closer look into o1-preview’s changes; it changed its answers in 12 out of the 40 questions (30%). Interestingly, on average, 3 out of 4 changes resulted in incorrect answers. In terms of the domains that o1-preview changed its answer at the most; from the highest percentage of questions changed in a category to the lowest: System based-practice and quality improvement (50%), Healthcare policy and economics (33%), Communication and interpersonal skills (32%), Patient safety (25%), Medical ethics and jurisprudence (22%). This notable difference in consistency showcases a vital advancement from o1-preview to o1. Both models performed highly; however, the latter had the confidence to stick to its answer while the latter did not, hindering its final score. Chance-corrected within-item self-agreement corroborated these observations. Cohen’s κ was 1.00 for GPT-4, GPT-4o, Gemini 1.5 Pro, and o1, indicating perfect self-agreement ((κ = 1). Whereas o1-preview showed only slight agreement (κ ≈ 0.02). The κ values therefore mirror the performance hierarchy and confirm that the “Are you sure?” probe did not artificially favor any model *(Supplementary Table 2.)*

## Discussion

Our findings contribute to ongoing AI-driven clinical practice and medical education discussions, aligning with prior work^18,27,28^, including Brin et al. (2023).

A key observation across these studies, which our findings also support, is the trend of newer LLMs outperforming their predecessors and typical human averages. This trend reflects the scaling laws observed in the papers published by the Open AI team, claiming consistent improvements in performance as models grow in compute and data exposure^23,24^. While it is not surprising that LLMs surpass the average non-specialist human in technical skills due to their extensive training. Their superiority in social or “soft” skills - as evident in our study, Brin et al.^18^, and Bicknell et al.^27^ - is surprising and challenges the assumption that these skills are exclusive human abilities. One possible counterargument is that, despite a correlational relationship^17^, performance on standardized exams may not necessarily translate into real-life outcomes and that discordances are possible. Despite the validity of the aforementioned counterargument, Ayers et al. (2023)^28^ study offers valuable evidence that supports our findings, albeit in a more realistic context. They performed a cross-sectional study of 195 patient queries from a social media forum, comparing responses from chatbots and licensed healthcare professionals, revealing that healthcare professionals rated chatbot responses higher in both quality and empathy, with chatbot replies being 9.8 times more likely to be categorized as empathetic or highly empathetic compared to those from human physicians^28^.

A noteworthy aspect of our study is the evaluation of Open AI’s new LLM, o1, which has not been previously studied in such a context. What is unique about o1 is that, unlike other LLMs that provide instantaneous answers, it utilizes a “chain of thought” reasoning process, breaking down problems step by step; similar to how humans solve complex issues^6,29^. This design helps enhance transparency by clarifying its decision-making process, thus addressing the common “black box” issue seen with many LLMs.

Like its design, o1’s results were unique. Our study was fortunate to test both versions, o1-preview, which we examined with other LLMs and o1 itself after its official release two months later. Both performed excellently despite the preview’s issue with overcorrection, which appears to have been resolved. Social skills questions require thought, intuition, and experience and may not find their exact answers in textbooks. Therefore, it is mechanistically plausible that LLMs optimized for chain-of-thought reasoning would perform best.

o1-preview had the potential to be the second-highest performer based on its initial accuracy (90%). However, its propensity to change its answers in response to skepticism resulted in a lower final score (77.5%). This contrasts with findings from a 2019 study by the National Board of Medical Examination (NBME) - the entity responsible for the USMLE examinations - that has found that out of over 27,000 Step 2 examinees, around two-thirds have changed their answers at least once. However, around two-thirds of those who changed their answers had higher scores for changing answers, and the remaining third had lower scores^30^. This goes against what was observed in o1-preview, where, on average, 3 out of 4 changes were to incorrect answers. While this initially suggested that reasoning models might be prone to overcorrection, o1 itself disproved this concern by showing perfect consistency across all questions. Thus, the overcorrection issue in the o1-preview version seems to be related to its early-stage status rather than a characteristic inherent to reasoning models.

Aside from o1, GPT-4o and Gemini 1.5 Pro, developed by different companies, performed equally well but displayed varied strengths in various categories. GPT-4o excelled in communication and interpersonal skills, which are essential for effective patient interactions. Gemini 1.5 Pro achieved perfect scores in medical ethics, system-based practice, and healthcare policy; crucial areas for navigating complex clinical environments. Such variability across soft skills categories has important implications for medical applications. It also suggests that, like humans, LLMs may have strengths and weaknesses, and optimizing for every skill and field of knowledge may not be the most efficient nor effective approach; instead, having different mini LLMs with specialized expertise may be a more cost-effective and time-efficient approach^31,32^. Taken together, these domain-level differences are most likely attributable to differences in each model’s training data and model architecture; details that private, for-profit companies rarely disclose in sufficient depth to permit firm interpretation. Consequently, a controlled head-to-head study, conducted with fuller technical transparency, will be required to establish which specific factors causally drive the observed gaps.

Our findings lead to several important takeaways. First, identifying the top-performing LLMs in social sciences in a clinical context offers valuable guidance for medical practitioners and educators considering LLM integration. Practical applications include clinical decision tools, patient communication chatbots, and simulations for evaluating social skills in virtual reality scenarios, potentially transforming how these skills are assessed. Second, o1, a new unexplored LLM with a unique design, has been examined and shows that in addition to its “exceeds human-PhD level” performance in science, technology, engineering, and mathematics (STEM) problems, according to OpenAI internal testing^22^, it also performs well in social skills questions, making it a well-rounded LLM. These takeaways impact AI in healthcare and encourage similar evaluations of social skills across AI models in other fields. Moreover, they offer insight into which methodological approaches lead to better social performance for AI scientists and developers.

Despite these notable positives, AI has drawbacks. Bias remains a significant concern, mainly when the training data reflect existing societal disparities. If the training data are biased, the output will likely follow suit. For instance, a study on a widely used health algorithm found that it discriminated against Black patients: at similar risk scores, Black patients were sicker than their White counterparts^33^. This highlights the risk of flawed model training and the need for careful evaluation when integrating such systems into healthcare.

Additionally, overreliance on AI for social and ethical decision-making may diminish a user’s ability to cultivate moral reasoning, judgment, and empathy. Over time, this may manifest as compromised independent thinking and problem-solving skills, particularly in high-stakes situations where AI tools may be unavailable or insufficient^34^.

There are some limitations to our study that need to be considered. First, our assessment tool is based on USMLE vignettes, which are written in English and calibrated to U.S. laws, ethics, and communication styles. Given that LLMs’ training datasets are disproportionately from English-speaking sources and Western cultural backgrounds^35–37^, and that LLMs have already shown a precedent of bias in matters such as gender^38^, nationality^39^, and religion^35^, all of this warrants the acknowledgment that our results should not be generalized to regions with different regulatory frameworks, cultural norms, or languages^40^. Therefore, regional context-specific evaluations and LLM fine-tuning are encouraged, and in some contexts, required, to ensure alignment with regional cultural, ethical, and legal standards. Second, It is important to consider the potential influence of data leakage, as LLMs may have been indirectly exposed to certain questions during pretraining^41^. Lastly, this study focused on a controlled question-answer format. Future research should explore how these AI models perform in more dynamic, real-world clinical environments where ethical decisions and interpersonal interactions are made in real-time. Third, despite utilizing a comparable number of social sciences questions as a typical USMLE Step 1 exam would have, it was not sufficiently powered identify significant between-LLM differences in performance. Larger item sets would better resolve modest gaps among top performers. Fourth, while our Cohen’s κ provided a chance-corrected, prompt-independent and within-item self-agreement between an initial answer and a final answer after the “are you sure?” skepticism-prompt; It does not constitute a formal psychometric reliability measure and may introduce some subjectivity. Future work should assess reliability via repeated, independent administrations and report complementary stability metrics.

## Supplementary Information

Below is the link to the electronic supplementary material.

Supplementary Material 1

Supplementary Material 2

## Data Availability

The data supporting the findings of this study are included as an appendix to this document. Supplementary Table 1 contains a comprehensive spreadsheet containing all the questions and corresponding answers utilized in the research; items removed for copyright reasons are not redistributed but are identified by their unique ID. Supplementary Table 2 presents the calculated Cohen’s κ statistics for the LLMs in table form. Supplementary Table 3 reports the inferential results for the accuracy tests (o1 vs. the 64% human benchmark: exact binomial; and o1 vs. the best non-reasoning LLM: Fisher exact), including Bonferroni-adjusted p-values. Supplementary Table 4 contains the short CSV used for inferential data-analysis. The annotated R script (LLM_socialskills_statistics.R) is used to generate Supplementary Tables 2–3 from the Supplementary Table 4.
